# Multilocus sequence and microsatellite identification of intra-specific hybrids and ancestor-like donors among natural Ethiopian isolates of *Leishmania donovani*

**DOI:** 10.1016/j.ijpara.2014.05.008

**Published:** 2014-09

**Authors:** Tesfaye Gelanew, Asrat Hailu, Gabriele Schőnian, Michael D. Lewis, Michael A. Miles, Matthew Yeo

**Affiliations:** a Serology Diagnostics and Research Laboratory, Centers for Disease Control and Prevention, Division of Vector-Borne Diseases Dengue Branch, San Juan, PR, United States; b Faculty of Medicine, Addis Ababa University, Addis Ababa, Ethiopia; c Institute of Microbiology and Hygiene, Charité University Medicine, Berlin, Germany; d Faculty of Infectious and Tropical Diseases, London School of Hygiene and Tropical Medicine, London, United Kingdom

**Keywords:** Hybridisation, *Leishmania*, MLMT, MLST, Recombination, Genetic exchange

## Abstract

Protozoan parasites of the genus *Leishmania* (Kinetoplastida: Trypanosomatidae) cause widespread and devastating human diseases. Visceral leishmaniasis is endemic in Ethiopia where it has also been responsible for fatal epidemics. It is postulated that genetic exchange in *Leishmania* has implications for heterosis (hybrid vigour), spread of virulent strains, resistance to chemotherapeutics, and exploitation of different hosts and vectors. Here we analyse 11 natural Ethiopian *Leishmania donovani* isolates consisting of four putative hybrids, seven parent-like isolates and over 90 derived biological clones. We apply a novel combination of high resolution multilocus microsatellite typing (five loci) and multilocus sequence typing (four loci) that together distinguish parent-like and hybrid *L. donovani* strains. Results indicate that the four isolates (and their associated biological clones) are genetic hybrids, not the results of mixed infections, each possessing heterozygous markers consistent with inheritance of divergent alleles from genetically distinct Ethiopian *L. donovani* lineages. The allelic profiles of the putative hybrids may have arisen from a single hybridisation event followed by inbreeding or gene conversion, or alternatively from two or more hybridisation events. Mitochondrial sequencing showed uniparental maxicircle inheritance for all of the hybrids, each possessing a single mitochondrial genotype. Fluorescence activated cell sorting analysis of DNA content demonstrated that all hybrids and their associated clones were diploid. Together the data imply that intra-specific genetic exchange is a recurrent feature of natural *L. donovani* populations, with substantial implications for the phyloepidemiology of *Leishmania*.

## Highlights

• Application of high resolution multilocus typing (MLMT and MLST) to characterise natural *L. donovani* parents and hybrids.
• Four isolates (and their associated biological clones) were found to be genetic hybrids, not the result of mixed infections.
• Markers were consistent with inheritance of divergent alleles from genetically distinct Ethiopian *L. donovani* lineages.
• Data imply that intra-specific genetic exchange is a recurrent feature of natural *L. donovani* populations.
• Uniparental maxicircle inheritance was shown for all the hybrids, each possessing a single mitochondrial genotype.

## Graphical abstract

**Figure:**
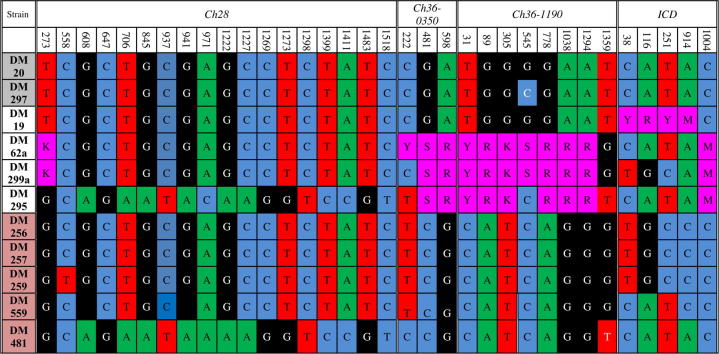

## 1 Introduction

The leishmaniases, caused by protozoan parasites of the genus *Leishmania* (Kinetoplastida: Trypanosomatidae), are worldwide vector-borne diseases transmitted by phlebotomine sand flies. *Leishmania* spp. infect a wide range of hosts including sylvatic and domestic animals. In humans, the leishmaniases are an enormous public health problem with a global prevalence of 12 million cases and a yearly incidence of 2 million cases (WHO, 2010). Infection with *Leishmania* can cause a broad spectrum of clinical presentations ranging from asymptomatic to simple cutaneous or destructive mucocutaneous lesions, or severe visceral leishmaniasis (VL) that is fatal without effective chemotherapy.

Historically, the population structure of *Leishmania* has been considered to be fundamentally clonal (Tibayrenc et al., 1993, Tibayrenc and Ayala, 2002) but with some limited historical evidence of recombination, for example, between *Leishmania braziliensis* and *Leishmania panamensis/guyanensis*, from multilocus enzyme electrophoresis (MLEE) and random amplification of polymorphic DNA (Belli et al., 1994, Banuls et al., 1997). Diversity observed within natural populations was thus generally attributed to the accumulation of mutations over time, with perhaps rare instances of genetic exchange. Nevertheless, evidence of genetic exchange between different *Leishmania* spp. in natural populations has been reported on several occasions (Belli et al., 1994, Kelly et al., 1991, Banuls et al., 1997, Ravel et al., 2006, Nolder et al., 2007, Hamad et al., 2011, Odiwuor et al., 2011). However, at the intra-species level, the identification of hybrids among natural populations has been more difficult and more equivocal due to the limited discriminatory power of methods such as MLEE. A few recent studies characterising natural *Leishmania* populations using highly polymorphic microsatellite markers have suggested the presence of genetic exchange (Chargui et al., 2009, Gelanew et al., 2010). In addition, Rougeron et al. (2011) described evidence of inbreeding in natural populations of both *Leishmania* (*Viannia*) *braziliensis* and *Leishmania* (*Viannia*) *guyanensis* based on linkage disequilibrium (LD), yet with a deficit of heterozygosity. The proposed reproductive strategy is therefore an alternating model of clonality in both the vertebrate and invertebrate hosts with non-obligatory sexual recombination in the sand fly. Whole genome sequencing of vector-isolated *Leishmania infantum* from southeastern Turkey supported the occurrence of hybridization and subsequent selfing; one of the parental genotypes was not identified but was likely to be from the *Leishmania donovani* complex (Rogers et al., 2014). The extant capacity of *Leishmania* spp. to undergo genetic exchange in the sand fly has been proven by generating experimental *Leishmania major* hybrids following co-passage of transgenic strains (Akopyants et al., 2009, Inbar et al., 2013). Hybrids isolated from the sand fly gut had genotypes consistent with meiosis. Furthermore, experimental crosses of fluorescent transgenic *L. donovani* in the sand fly produced dual expression in single cells, consistent with intra-species genetic exchange (Sadlova et al., 2011).

Genetic exchange has potential implications for heterosis (hybrid vigour), the emergence and spread of virulent strains, resistance to chemotherapeutics, exploitation of different hosts and vectors, and adaptation to new ecological niches that may provide a selective advantage. For example, a startling observation by Volf et al. (2007) provided clear evidence that *L. infantum/major* hybrids possess enhanced transmission potential. *Leishmania infantum* is not normally able to infect the broad ranging *Phlebotomus papatasi* but, remarkably, the resultant hybrids were able to do so with potentially profound epidemiological consequences. *Leishmania braziliensis*/*Leishmania peruviana* hybrids have also been implicated as agents of destructive forms of mucocutaneous leishmaniasis (Nolder et al., 2007).

The work described here was designed to accomplish three main objectives applied to a panel of 11 natural isolates of north Ethiopian origin: (i) to characterise four putative hybrids using a range of high resolution markers; (ii) to identify likely parental genotypes among seven north Ethiopian *L. donovani* representatives that did not have hybrid-like microsatellite genotypes; and (iii) to discount the possibility that hybrid-like microsatellite genotypes were the result of mixed infections. We applied a novel combination of multilocus sequence typing (MLST) targets and highly resolutive multilocus microsatellite typing (MLMT) markers (Kuhls et al., 2007, Gelanew et al., 2010) in conjunction with mitochondrial (maxicircle) sequencing and DNA content analysis. We demonstrate that four natural isolates are bona fide hybrids and not the result of mixed infection. The likely parental origins and epidemiological implications are discussed.

## 2 Materials and methods

### 2.1 Parasite strains and DNA isolation

**Fig. 1:**
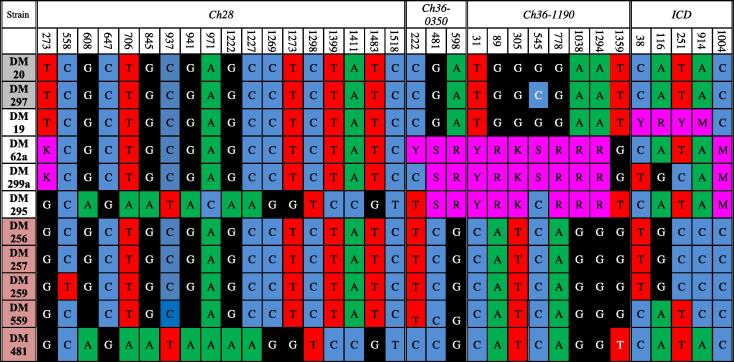
Summary of single nucleotide polymorphism distributions across four multilocus sequence typing loci (Chromosome (*Ch*)*28*, *Ch36-0350*, *Ch36-1190* and isocitrate dehydrogenase (*ICD*)) in *Leishmania* clones. Data from the glucose-6-phosphate isomerase (*GPI*) locus is not shown (all sequences were identical). Isolate names are abbreviated with World Health Organization strain codes shown in Table 1. DM19, DM62, DM295 and DM299 hybrids possess heterozygous single nucleotide polymorphisms. DM20 and DM297 (in colour) represent parent-like A donors and DM256, DM257, DM259, DM481 and DM559 (in colour) represent possible parent-like B donors. Full IUPAC codes for heterozygote single nucleotide polymorphisms are as follows: K(G,T); M(A,C); R(G,A), S(G,C); Y(T,C). Numbers indicate the nucleotide position within the loci. (For interpretation of the references to colour in this figure legend, the reader is referred to the web version of this article.)

A panel of 11 north Ethiopian *L. donovani* isolates was available, originating from a population of Ethiopian isolates containing some putatively characterised as hybrids by Gelanew et al. (2010), participants were enroled according to written informed consent procedures and approved by the Institutional Review Boards of the Faculty of Medicine, Addis Ababa University, Addis Ababa. Full World Health Organization (WHO) isolate labels are shown in Table 1 and isolate characteristics are shown in Supplementary Table S1. Isolates that were available comprised four putative hybrids, DM19, DM62, DM295 and DM299. Two of the hybrids were isolated from an HIV-infected patient; DM62 and DM299 were isolated from the same HIV/VL co-infected patient during different episodes of the disease. Parent-like representatives were DM20, DM297 (designated hypothetical parent group A), and DM256, DM257, DM259, DM481, DM559 (designated hypothetical parent group B, Fig. 1, Table 1). A total of 90 biological clones were generated, a maximum of 10 per isolate (DM19, eight clones; DM62, 10 clones; DM256, nine clones; DM257, nine clones; DM259, nine clones; DM295, nine clones; DM 297, nine clones; DM299, eight clones; DM481, nine clones; DM599, 10 clones). *Leishmania* were cultured in supplemented RPMI liquid medium at 28 °C, as previously described (Mauricio et al., 2004). Isolates were then cloned on solid media as described by Yeo et al. (2007). Once single colonies became visible they were removed and inoculated into liquid culture medium. DNA was isolated from each clonal culture using DNA isolation kits (Promega, UK) following the manufacturer’s instructions.

**Table 1:** Microsatellite profiles at five loci of hybrids and their corresponding putative parents for *Leishmania* isolates.

	Strain WHO code	Marker				
		Li41-56	Li46-67	Li22-35	Li71-33	Li71-7
Hypothetical parent A (*n *= 2)	MHOM/ET/2007/DM20	89/89	72/72	78/78	99/99	92/92
	MHOM/ET/2008/DM297	83/83	70/70	78/78	113/113	92/92

Hybrid isolates (*n *= 4)	MHOM/ET/2007/DM19^a^	91/91	70/72	78/98	99/103	92/92
	MHOM/ET/2007/DM62	89/91	70/72	78/98	99/103	90/92
	MHOM/ET/2008/DM295	89/91	70/72	78/98	99/103	90/92
	MHOM/ET/2008/DM299	89/91	70/72	78/98	99/103	90/92

Hypothetical parental B (*n *= 5)	MHOM/ET/2008/DM256	91/91	72/72	98/98	117/117	92/92
	MHOM/ET/2008/DM257	91/91	72/72	98/98	117/117	92/92
	MHOM/ET/2008/DM259	83/89	72/72	98/98	117/117	92/92
	MHOM/ET/2009/DM481	89/89	72/72	98/98	113/113	92/92
	MHOM/ET/2009/DM559	83/89	72/72	98/98	113/113	90/92

**Fig. 2:**
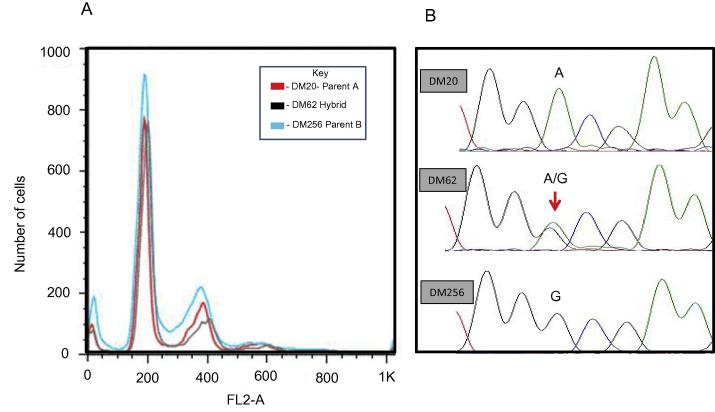
Flow cytometric analysis and sequencing electropherograms. **(**A) Flow cytometric analysis of relative DNA content in *Leishmania* clones. Data is plotted as a FL2 area histogram with gates were created for G1-0 (2*n*) peaks and for G2-M (4*n*) peaks and overlaid DNA histograms illustrating comparable (2*n*) DNA content in hybrid and parent-like isolates. (B) Electropherograms showing two variable single nucleotide polymorphisms present in homozygous non-hybrids (DM20, DM256) and the equivalent bi-allelic locus present in hybrid DM62 at the same locus.

WHO, World Health Organization.

a Hybrid DM19 is heterozygous at three loci unlike DM62, DM295 and DM299, which are heterozygous across all five loci, as is congruent with multilocus sequence typing (MLST) data (Fig. 2).

### 2.2 MLMT

Five microsatellite markers were selected (Table 2) based on their ability to distinguish putative hybrids from parents (Gelanew et al., 2010). Fluorescent-conjugated forward and non-conjugated reverse primers (Eurofins, Germany) were used for amplification. The amplification conditions and reaction cycles were performed as described by Ochsenreither et al. (2006) with slight modifications. After an initial denaturation step at 95 °C for 10 min, samples were processed through 35 cycles consisting of 95 °C for 30 s, annealing for 30 s at the temperature (*T_A_*) indicated in Table 2 and 72 °C for 1 min, followed by a terminal elongation step at 72 °C for 10 min. Each reaction was performed in a final volume of 20 μl consisting of 1× ThermoPol reaction buffer (New England Biolabs (NEB), UK), 1.5 mM MgCl_2_, 0.2 mM dNTPs, 10–25 pmol of each primer, 1 unit of *Taq* polymerase (NEB, UK) and 20–30 ng of genomic DNA. Allele sizes were estimated using an automated capillary sequencer (ABI3730, Applied Biosystems, UK) and were checked manually.

**Table 2:** Properties of microsatellite markers used in this study.

Marker	*T_A_* (°C)	Chromosome	Forward (5′–3′)	Reverse (5′–3)
Li22-35	52	1	CTTGATGTTCGGGTTAGCAAG	ATGCACACCAAAAATCATGTG
Li41-56	50	36	TTGCTTCATGATAACAACTTGG	CCTGTTGGTGTGAGTTCGTG
Li46-67	50	31	TCTTCTTTCGTTAGCTGAGTGC	CTGTATCACCCATGAGGGGC
Li71-7	50	30	GCTGCAGCAGATGAGAAGG	GTGAGAAGGCAGGGATTCAA
Li71-33	50	31	CTCCTTTCACACCCGCCTCT	GAGAGAAGACGAGCCGAAGT

*T_A_*, temperature of annealing.

### 2.3 MLST

MLST markers were amplified from genomic DNA extracted from the isolates and biological clones. Table 3 summarises PCR amplification primers and annealing temperatures. Genetic markers consisted of four coding nuclear markers (glucose-6-phosphate isomerase, *GPI*; isocitrate dehydrogenase, *ICD* ; *Ch28; Ch36-1190*) and one non-coding nuclear marker (*Ch36-0350*). Targets were chosen following screening of a panel of markers known to exhibit sequence diversity within the *L. donovani* complex on the basis of GenBank sequences. Amplifications were performed with an initial denaturation step at 94 °C for 3 min, followed by 30 cycles of 94 °C for 30 s, with annealing and elongation steps for each marker as described herein, and a final elongation step at 72 °C for 10 min. The annealing temperatures for *GPI* and *ICD* were 57 °C and 61 °C, respectively, for 90 s and for *CH28-0190, CH36-1130 and CH36-0350* were 53 °C, 59 °C and 61 °C, respectively, for 60 s. Each reaction was performed in a 20 μl total volume containing: 20 ng of genomic DNA, 20 pmol of each primer, 2 mM dNTPs, 1.5 mM MgCl_2,_ and 5 U *Taq* (BIO-21086, Bioline, UK). PCR products were visualised on 1.5% agarose gels and the appropriate bands excised and purified using a QIAGEN Gel Extraction Kits (Qiagen, UK) or SureClean (Bioline). Bi-directional sequencing was performed, using internal primers where required (Table 3), with Big Dye Terminator Cycle Sequencing V3.1 (Applied Biosystems) and an ABI PRISM 377 DNA Sequencer (Applied Biosystems) according to the manufacturer’s protocols. Sequence data were assembled manually in BioEdit v7.0.9.0 (Ibis Biosciences, USA) and ambiguous peripheral regions of aligned sequences were discarded to produce unambiguous partial gene sequences for each isolate or clone. Chromatograms were examined visually in both directions for the presence of heterozygous bi-allelic single nucleotide polymorphisms (SNPs) at a single locus (Fig. 2). Re-sequencing was undertaken if results were ambiguous. SNP differences between isolates and clones were recorded and tabulated together with their associated positions (Fig. 2).

**Table 3:** Multilocus sequence typing targets for *Leishmania donovani*.

Locus	Chromosome number	No. of polymorphic sites/SNPs	Primer sequences (5′–3′)	Annealing temp (*T_A_* °C)	bp
GPI^a^	12	0	F:GACCGAGGCACTTGAAG R6: TGAATGAGCTGGTAGAATG	57	1150
ICD^a^	10	5	F:ATGTTCCGCCATGTTTCGG R:TTACGCGCTCATCGCCTT	61	1267
Ch28^a^	28	18	INTF:GTCGCAGTCCAACTCCCATA INTR:CGCATAGCAAAAGCCAAA	53	1530
Ch36-1190^a^	36	8	F:GCTTCTCGCTATTGCTCGTC R:ACTGGCAGGCACACATCAG INTFA: GCGGCTACCTCGCCCTCAGT INTFB:GTGAAGGACCAAGCTGCCTGG INTRA;TGTGAAGCACCAGCAGGACGG	59	1640
Ch36-0350^b^	36	3	F:ACTTGGTCTTGGTACGG R:TGGAGGACGGAGAGACTTTG INTF:GTGAATGGAGGGCAGACG INTR:GTCGTGAAAAGCGAGAAGGT	61	850

SNP, single nucleotide polymorphism; *T_A_*, temperature of annealing; *GPI,* glucose-6-phosphate isomerase; *ICD,* isocitrate dehydrogenase; *Ch*, chromosome.

a Coding nuclear marker.

b Non-coding nuclear marker.

### 2.4 Mitochondrial maxicircle gene sequencing (mMLST)

The cytochrome b gene (*CYTb*) was amplified and sequenced using primer pairs LCBF: GGTGTAGGTTTTAGTTTAGG and LCBR2: CTACAATAAACAAATCATAATATACAATT (Luyo-Acero et al., 2004). Cycle conditions consisted of an initial denaturation at 94 °C for 30 s followed by 35 cycles of denaturation at 94 °C for 30 s, annealing at 50 °C for 30 s, extension at 68 °C for 5 min and a final extension step at 72 °C for 7 min. The amplified fragments were sequenced in both directions using the same set of primers and following the sequencing methods described above.

### 2.5 DNA content analysis

Approximately 1 × 10^7^ cloned *L. donovani* parasites were harvested from mid-log phase liquid cultures. Parasites were pelleted by centrifugation at 3,000*g* (20 min), washed twice in ice-cold PBS and fixed with 100% methanol for 48 h at 4 °C. Two subsequent washes with ice-cold PBS were performed, after which cells were resuspended in PBS to a final density of 1 × 10^6^ cells/ml. Propidium iodide and RNAse A were added to a final concentration of 10 μg/ml and the mixture incubated for 45 min at 37 °C in the dark. Fluorescence was detected using a FACSCalibur flow cytometer, on channel FL2. For each sample a minimum of 10,000 events was counted. For each strain/clone, two independent assays were performed. Data analysis used FlowJo software (Tree Star Inc., Oregon, USA). Gates were created for G1-0 (2*n*) peaks and for G2-M (4*n*) peaks. Mean G1-0 values were taken to infer relative DNA content. Relative DNA content values were calculated as a ratio in comparison with an internal standard (*L. major,* Friedlin strain). For putative hybrids, the ratios relative to each putative parent (A or B) were also recorded.

## 3 Results

### 3.1 MLMT hybrids, parental genotypes and exclusion of mixed infections

Uncloned stocks from Ethiopia had identified a subset of putative hybrids possessing heterozygous profiles, with MLMT genotypes composed of alleles that were otherwise restricted to two distinct *L. donovani* populations in northern Ethiopia (Gelanew et al., 2010). Heterozygous genotypes could have been the result of amplification of multiple alleles from multiclonal sources. We therefore prepared a maximum set of 10 biological clones for each of the 11 selected strains and conducted MLMT analyses. MLMT profiles across five loci are summarised in Table 1. Overall in the four putative hybrids, MLMT analyses of clones showed high levels of heterozygosity that were not a result of multiclonality, in stark contrast with the high levels of homozygosity in putative parents (group A and group B). In more detail, the two non-hybrid (group A) strains and all of their derived clones were consistently homozygous across the five microsatellite markers. Similarly, the five non-hybrid (group B) strains and derived clones were generally homozygous across the five loci, with the exception of the heterozygous genotypes DM259 at locus Li41-56 and DM559 at loci Li41-56 and Li71-7. Three of the four hybrid strains (and their biological clones) were heterozygous across all five loci. The exception was DM19, which was heterozygous in three loci (Li46-67, Li22-35 and Li71-33) and homozygous at Li41-56 and Li71-7. Thus the heterozygous alleles in the five hybrid microsatellite loci matched with corresponding homozygous alleles among the parent-like isolates. However there were four examples where the origins of donor alleles in the putative hybrids could not be determined. As shown in Table 1, these were: (i) the 103 bp allele at the heterozygous Li71-33 site in the hybrids was absent from both putative parental groups; (ii) for the heterozygous loci at Li41-56 (89/91), Li46-67 (70/72) and Li71-7 (90/92) in the hybrids one allele, 89, 72 or 92, respectively, could be found among both parental groups; and (iii) at locus (Li71-7), one isolate among parental group B was heterozygous (90/92). An exception was also seen at a single site for the MLST target *ICD* (see Section 3.2). Taken alone, microsatellite data were less robust than MLST data (Section 3.2) in identifying likely parental donors.

### 3.2 MLST comparisons reveal hybrids and putative parents

Although MLMT data detected consistent hybrid profiles, allele sharing across hypothetical non-hybrid groups (A and B) meant that inferring likely parental multilocus genotype donors was not possible. We therefore sought to analyse nucleotide sequences from several nuclear loci, reasoning that patterns of SNP marker inheritance would be more discriminatory. Gene identities (IDs) were confirmed by BLAST searches and submitted to TriTypDB 4.2 (http://TriTrypDB.org) to determine chromosomal locations. Locations for each of the nuclear gene fragments are shown in Table 3. Two gene fragments (*Ch36-0350 and Ch36-1190*) were located on chromosome (Ch)36 (P:95153–94109 and P:429635–431277, respectively). The remaining genes were on independent chromosomes 10, 12 and 28. Nucleotide sequence data reported in this paper are available in the GenBank™ database under the Accession Nos. KJ907394- KJ907448.

We characterised a total of 90 biological clones originating from both the putative hybrids (four isolates) and parent-like strains (seven isolates). The *GPI* locus was monomorphic and homozygous across all 11 strains and for the 90 biological clones; *GP*I was therefore not included in subsequent analysis. A summary of the distribution of SNPs and their relative positions in the remaining four loci is shown in Fig. 1. A total of 34 unambiguous SNP positions were detected across all loci. Locus *Ch28* was the most diverse fragment with 18 SNPs, followed by *Ch36-1190* with eight, *ICD* with five and *Ch36-0350* with three. Heterozygous sequence profiles were found only in the hybrids; 16 SNPs displayed bi-allelic heterozygous profiles in at least one isolate: locus *Ch28* with one such heterozygous site, *Ch36-1190* with seven, *ICD* with five and *Ch36-0350* with three. In contrast the putative parent-like isolates (A and B) were homozygous across all SNP positions. The high number of heterozygous sites seen in the hybrids, in comparison with the relative rarity of heterozygosity in the putative parents, is consistent with the patterns of heterozygosity and homozygosity seen in the MLMT data.

Each of the four hybrid strains had a unique multilocus genetic profile. Interestingly, even hybrid isolates DM62 and DM299, which were isolated sequentially from the same patient, had distinct *Ch36-0350* and *ICD* sequences, with one and four SNP differences, respectively, suggesting either multiclonality in this patient or clonal diversification in vitro.

Variable sites were then examined with respect to their presence/absence in strains and clones from the hybrid group (Fig. 1). Three classes of marker distribution were observed. In some cases SNP distribution in hybrid group strains was consistent with acquisition of a group A and a group B allele, for example, DM62, DM299 and DM295 had heterozygous sequences compatible with inheritance of an A-like and a B-like *Ch36* sequence. In other cases the hybrid group strains were homozygous for a group A-like or a group B-like sequence, for example, DM19 for the *Ch28* and *Ch36* loci and DM299 for the *ICD* locus. There were also several instances where the sequences in hybrid group strains were predominantly homozygous for an A-like or B-like sequence but with clear heterozygosity at the extreme 5′ and/or 3′ regions, e.g. DM62 and DM299 had *Ch28* sequences that were homozygous for A-like SNPs at all informative positions except the most 5′ site (position 273) for which it had both an A-like and B-like SNP. Finally, both heterozygous sites for *ICD* in DM62, DM295 and DM299 were not present in non-hybrid groups A and B and were not informative with respect to inheritance. Thus, although it is clear the MLST data overall support the hypothesis that the hybrid group strains are derived from a hybridization event between group A and group B, the distribution of specific markers suggests additional complexity that may be a result of several post-hybridization events including loss of heterozygosity due to gene conversion or subsequent rounds of hybridization (inbreeding), or that the diversity within groups A and B may not have been sampled adequately.

We next examined the hybrid and parental strains in order to identify instances of strain-specific marker inheritance. There was only one intra-group SNP in non-hybrid group A (*Ch36-1190*, position 545, G/C); the ‘G’ allele was uniquely present in group A strain DM20 and three of the hybrid group strains, DM19, DM62 and DM299 (Fig. 1). This indicates that DM20 is a more similar candidate A parent of these hybrids than DM297. There was a greater degree of intra-group variation in non-hybrid group B. The group B-like *Ch28* and *ICD* alleles present in hybrid group strain DM295 were clearly most closely related to the group B strain DM481. Where informative markers were present for hybrid group strains DM62, DM299 and DM19, the sequences indicated higher identity with group B strains DM256, DM257 and DM259 than DM481 or DM559. The high number of SNP differences separating the different likely B-like allele donors for DM295 and DM62, DM299 and DM19 suggest two independent hybridization events, although a single event followed by significant subsequent diversification cannot be ruled out. It is also possible that DM19 stems from a third independent event, particularly given its distinct MLMT profile.

In summary the MLST data showed a pattern of marked but not fixed heterozygosity in the hybrids in comparison with the rarity of heterozygosity in non-hybrid group strains, similar to that observed for the MLMT data. Taken alone, microsatellite data are less robust than MLST data in identifying likely parental contributors. But taken together, both the MLMT and MLST data indicate overwhelmingly that strains DM19, DM62, DM295 and DM299 are genetic hybrids. The distribution of SNP markers allowed some tentative parent-hybrid relationships to be identified and indicated the hybrid group strains have either three independent origins or, less likely, a single origin with extensive subsequent divergence.

### 3.3 Kinetoplast DNA (kDNA) maxicircle-encoded CYTb sequences support uniparental mitochondrial inheritance

SNP markers were identified in a region of the *CYTb* gene to determine inheritance patterns of the mitochondrial maxicircles. Six SNPs were identified spanning a 748 bp fragment of the *CYT*b locus. Relative positions were p19 (G, T), p350 (A, T), p499 (G, A), p566 (A, C), p583 (A, G) and p649 (G, A). Two maxicircle genotypes were present among the isolates and clones studied here, with either TTACGA or GAGAAG SNP profiles across the six SNP sites. All hybrids and derived hybrid clones (a minimum of four clones per strain) had the type GAGAAG SNP profile, consistent with uniparental inheritance of the maxicircle DNA, in contrast to the biparental inheritance of the nuclear markers. There were no other combinations of maxicircle profiles and there was no heterozygosity. The GAGAAG maxicircle profiles found in the hybrid group strains were identical in both non-hybrid A strains (DM20, DM297) and in group B strains (DM559, DM481). Within group B, both mitochondrial genotypes were present.

### 3.4 Flow cytometry measurement of cellular DNA content

An example of the DNA content determinations on fixed parasites of hybrid and parents, stained with the fluorescent DNA binding dye propidium iodide, is shown in Fig. 2. A reference diploid (*2n*) strain was included in each run and duplicate runs were performed. All isolates produced clear G1-0 (*2n*) and G2-M (*4n*) fluorescence peaks. There was no significant difference in relative DNA contents between any of the hybrid or non-hybrid strains. The results are a strong indication of diploidy in all clones that were analysed.

## 4 Discussion

Here we provide evidence for bona fide hybrid strains and ancestral-like genotypes selected from a natural population of *L. donovani* causing VL in northern Ethiopia. Our evidence is based on high resolution MLMT and novel MLST targets applied to the nuclear genomes of 11 north Ethiopian *L. donovani* isolates and 90 clones derived from those. Importantly, the genotypes of all of the clones corresponded with the genotypes of the original isolates, and these results were therefore not confounded by artefactual analysis of isolate mixtures.

By comparing genetic profiles of the north Ethiopian isolates and their derivative clones, four of the isolates were shown to be hybrids. The hybrids were heterozygous at both MLST and MLMT nuclear loci, with significant corresponding homozygosity present among putative parental strains of groups A and B. Breakpoints within individual alleles were not detected when applied to recombination algorithms (RDP V4), suggesting the inheritance of a discrete allele from each of the parents.

Strikingly, the heterozygosity in the MLST data from hybrids almost precisely matched the homozygous SNPs at the same sites in the deduced pairs of parents. Results were mirrored in the MLMT analysis, although the technique is less robust and not suitable to fully resolve parents and donors using the five loci. However, together MLST analysis and MLMT spanning nine loci proved to be powerful and complementary techniques for identifying hybrids of *L. donovani*. As such, the novel combination of markers may be useful for screening whole populations for hybrids when sequencing is not feasible.

Not all of the four *L. donovani* hybrid isolates were identical and there were varying degrees of SNP differences between them. Two hybrids (DM62 and DM299) had very similar genetic profiles, possibly originating from the same event. Remaining hybrids (DM295 and DM19) were more distinct. Our data show that DM62 and DM299 appear to possess a different parent (B) allele than DM295 and we consider this suggests more than one recombination event. Hybrid DM19 was an unusual outlier exhibiting reduced heterozygosity, relative to the other hybrids, yet still retaining a hybrid signal in one MLST locus and two MLMT loci. One interpretation of these data is another recombination event, although long-term divergence from hybrids (DM62 and DM299) or divergence via gene conversion cannot be entirely excluded. Recent whole genome sequencing of 11 *L. infantum* isolates from a focus of cutaneous leishmaniasis (CL) in southeastern Turkey (Rogers et al., 2014) suggested that they derived from a single cross of two diverse strains followed by recombination within the population. Although one of the parental types was not present, it was likely to be from the *L. donovani* complex. They concluded that recombination in *L. infantum* is probably rare in the field as judged by the length of parental sequence blocks, and the pattern of linkage between SNPs. Concerning our data, the allelic contributions from both parental types were present and appear relatively conserved, also suggesting recent genetic exchange events. However, further sampling would be required at the population level to resolve these processes and to assess more accurately the presence and frequency of endogamy which, at high frequencies, would be characterised by a trend towards homozygosity within a population (Rougeron et al., 2011).

Not surprisingly, the genotypes considered here to be parental did not correspond precisely with those of predicted parents; for example one of the SNPs at the heterozygous *ICD* locus is missing from the putative parents. There are a number of explanations for differences between the hybrids and putative parent-like isolates. Firstly, it is most likely that multiple other genotypes circulating naturally were not sampled and some of these may be even more similar to the predicted parents. Secondly, some gene conversion or mutation is inevitable with time since hybridization, restoring homozygosity or generating divergent genotypes at some loci. Differences between the hybrids could be also accounted for by repeated selfing, although the largely corresponding heterozygous SNPs and corresponding homozygous “donors” in MLST and MLMT outputs make this unlikely.

We also investigated the *L. donovani* maxicircle kDNA inheritance patterns by sequencing the *CYTb* locus. Several SNPs in *CYTb* were identified. Identified SNPs formed two distinct mitochondrial marker combinations among the non-hybrid *L. donovani* isolates and clones. As expected, for haploid mitochondrial markers no heterozygosity was observed. We found that all four hybrid isolates and their derivative clones only inherited a single maxicircle DNA genotype. Furthermore, this was the mitochondrial type genotype of DM20, one of the most likely parent-like genotypes of all four hybrids. Thus, we find uniparental inheritance of mitochondrial DNA. This accords with mitochondrial inheritance of the experimental hybrids of *L. major*, in which 12 of 18 hybrids inherited the mitochondrial genotype of one parent and six inherited the genotype of the other parent (Akopyants et al., 2009).

We determined the DNA contents of the hybrids relative to the putative parents by fluorescence activated cell sorting (FACS analysis). The FACS analysis demonstrated that hybrids, non-hybrids and resultant clones were all diploid. In contrast, stable hybrid aneuploids have been described in *L. major* following extensive serial passage in vitro (Akopyants et al., 2009, Inbar et al., 2013). Reversion to diploidy over longer time periods cannot be ruled out and has been observed with aneuploid genomes that are a product of parasexual processes. One hybrid tetraploid clone of *L. major*, stable in vitro and passaged through mice, resulted in the recovery of diploids (Inbar et al., 2013). Although there is striking synteny at the gene content level between different *Leishmania* genomes, there is extensive variation in some at the level of chromosomes (Downing et al., 2011) and the characteristics of recombination in *L. donovani* may not be identical to that seen in *L. major.*

On balance, the hybrids observed here among the north Ethiopian population of *L. donovani* appear compatible with products of meiosis in that: there are high levels of heterozygosity relative to the parents, with clearly identifiable donor SNPs; one homologue is present from each parent and recombinant genotypes are observed across multiple loci, consisting of nuclear and microsatellite markers; there is uniparental maxicircle inheritance, and a majority 2*n* ploidy. The routine generation of experimental hybrids would be needed to fully address the nature of recombination in *L. donovani* and whether hybridization follows the pattern of orthodox meiosis.

It is interesting to note that two of the reported isolates (Dm62 and Dm299) were from a single HIV co-infected patient during different episodes of the disease in northern Ethiopia. Although the two hybrids were similar, SNP differences were apparent. The presence of multiple genotypes in a single severely immunocompromised patient may increase the likelihood of recombination between genotypes within the vertebrate host (Ravel et al., 2006). Alternatively, immunocompromised individuals may be more susceptible to infection with a wider range of genotypes from a sand fly vector in which genetic exchange has occurred, as is supported by the experimental proof of capacity for genetic exchange in the sand fly. Additionally, an immunocompromised status may enable less fit hybrids to persist when they would otherwise be cleared from immune competent individuals (Miles et al., 2009).

The full consequences of genetic exchange in *L. donovani* are as yet uncertain. For example, do the hybrids described here also differ in their ability to exploit different hosts and vectors or cause differential pathology in humans? The *L. donovani* hybrid and parental isolates characterised provide an excellent opportunity for follow up analysis and for further experiments on hybridization, the mechanisms of genetic exchange and the associated generation of phenotypic diversity.
