# Supplementary material for: Multilocus sequence and microsatellite identification of intra-specific hybrids and ancestor-like donors among natural Ethiopian isolates of Leishmania donovani
Source: Int J Parasitol. 2014 Sep;44(10):751–7. doi: 10.1016/j.ijpara.2014.05.008 (PMC4147965; doi:10.1016/j.ijpara.2014.05.008)
Supplement: Supplementary data 1 [file mmc1.doc]

**Supplementary Table S1.** Characteristics *of Leishmania donovani* strains used in this study.

| **WHO code** | **Country** | **District/Travel history** | **Pathology** | **Population**^b^ | **Source** |
| --- | --- | --- | --- | --- | --- |
| MHOM/ET/2007/DM19 | Ethiopia | Gondar | VL | NE/SD | Gelanew et al., 2010 |
| MHOM/ET/2007/DM20 | Ethiopia | Gondar | VL | NE/SD | Gelanew et al., 2010 |
| MHOM/ET/2007/DM62^a^ | Ethiopia | Libo Kemkem-Abdurafi | VL/HIV+ | NE/SD | Gelanew et al., 2010 |
| MHOM/ET/2008/DM256 | Ethiopia | Military-suspected NE endemic | VL/HIV+ | NE/SD | Gelanew et al., 2010 |
| MHOM/ET/2008/DM257 | Ethiopia | Gondar-Humera | VL/HIV+ | NE/SD | Gelanew et al., 2010 |
| MHOM/ET/2008/DM259 | Ethiopia | Bihar Dar-Humera | VL | NE/SD | Gelanew et al., 2010 |
| MHOM/ET/2008/DM295 | Ethiopia | Belessa | VL | NE/SD | Gelanew et al., 2010 |
| MHOM/ET/2008/DM297 | Ethiopia | Libo Kemkem-Abdurafi relapse of DM-62 | VL/HIV+ | NE/SD | Gelanew et al., 2010 |
| MHOM/ET/2008/DM299^a^ | Ethiopia | Libo Kemkem-Abdurafi relapse of DM-62 | VL/HIV+ | NE/SD | Gelanew et al., 2010 |
| MHOM/ET/2009/DM481 | Ethiopia | Libo Kemkem | VL | NE/SD | Gelanew et al, 2010 |
| MHOM/ET/2009/DM559 | Ethiopia | Gondar | VL  HIV status | NE/SD | Gelanew et al., 2010 |

WHO, World Health Organization; VL, visceral leishmaniasis.

^a^ isolated from the same patient with different episodes of VL.

^b^Population NE/SD contained strains from northwest Ethiopia and Sudan.

**References**

Gelanew, T., Kuhls, K., Hurissa, Z., Weldegebreal, T., Hailu, W., Kassahun, A., Abebe, T., Hailu, A., Schonian, G., 2010. Inference of population structure of *Leishmania donovani* strains isolated from different Ethiopian visceral leishmaniasis endemic areas. PLoS Negl. Trop. Dis., 4, e889.
